# Affinity-Based Methods for Site-Specific Conjugation of Antibodies

**DOI:** 10.1021/acs.bioconjchem.1c00313

**Published:** 2021-08-09

**Authors:** Emma von Witting, Sophia Hober, Sara Kanje

**Affiliations:** Department of Protein Science, School of Engineering Sciences in Chemistry, Biotechnology and Health, AlbaNova University Centre, SE-114 19, Stockholm, Sweden

## Abstract

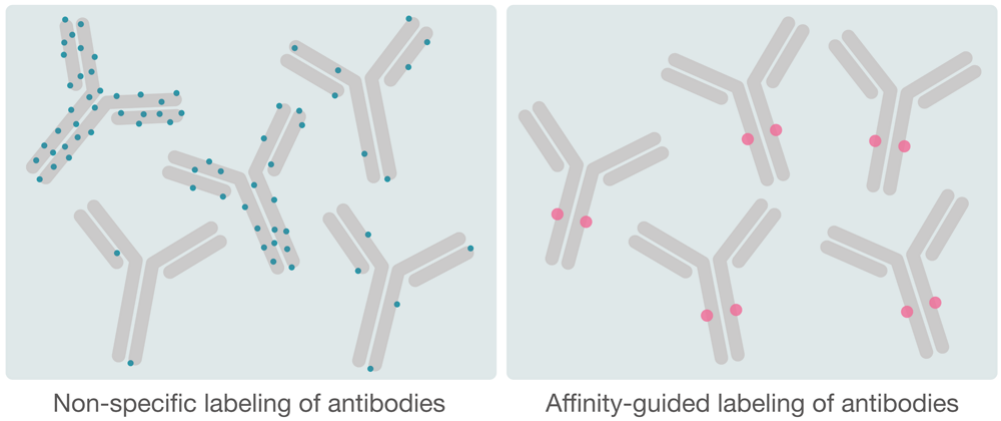

Conjugation of various reagents to antibodies has long been an elegant way to combine the superior binding features of the antibody with other desired but non-natural functions. Applications range from labels for detection in different analytical assays to the creation of new drugs by conjugation to molecules which improves the pharmaceutical effect. In many of these applications, it has been proven advantageous to control both the site and the stoichiometry of the conjugation to achieve a homogeneous product with predictable, and often also improved, characteristics. For this purpose, many research groups have, during the latest decade, reported novel methods and techniques, based on small molecules, peptides, and proteins with inherent affinity for the antibody, for site-specific conjugation of antibodies. This review provides a comprehensive overview of these methods and their applications and also describes a historical perspective of the field.

## Introduction

Antibodies have a long-standing reputation as excellent tools in many medical and biological applications because of their capacity to selectively bind to specific target molecules with high affinity. Both in the diagnostic and in the therapeutic fields, antibodies are commonly decorated with specific active groups, either to make them detectable or to equip them with a specific characteristic or activity. The added characteristic could be a group that would be useful in a diagnostic setup, such as a fluorescent or radioactive label or an enzyme that can be used for detection. By adding such labels to an antibody, it can be used in a variety of analytical or diagnostic methods such as immunohistochemistry (IHC), enzyme-linked immunosorbent assay (ELISA), or fluorescence-activated cell sorting (FACS). Furthermore, the number of therapeutically used monoclonal antibodies conjugated to small therapeutic molecules, so-called antibody–drug conjugates (ADC), is continuously increasing and with that comes a demand for efficient, stable, and selective conjugation strategies.^1,2^ When developing an ADC, it is important that the conjugation is efficient, stable, and uniform to provide a safe and effective therapy. This is however not always the result when using traditional conjugation methods. Therefore, many novel methods for conjugation have been developed during recent decades,^3^ some of which will be the focus of this review.

The traditional, and also most widespread, methods developed for protein conjugation are nonspecific and based on the utilization of side chains of frequently appearing amino acids, such as thiols, carboxyl groups and, most commonly, primary amines found in the N-terminus of the protein and on the side chain of lysines.^4^ These conjugation methods can pose problems since it is difficult to tune the labeling, as neither the position nor the exact number of labels per antibody can be controlled. Meanwhile, several studies have established the importance of homogeneous conjugates for increased therapeutic potential of ADCs.^5,6^ For technical and diagnostic antibodies, unspecific labeling with fluorescent dyes might become problematic due to clustering and quenching of the fluorescence if several labeling molecules end up in close proximity.^7^ Furthermore, lysines are often located in protein binding surfaces due to their positive charge. If regions close to, or within, the paratope of the antibody contain lysines, the conjugated moiety may interfere with the antibody’s capability to interact with its antigen. This might impair the efficacy of the antibody and thus give a less efficient therapeutic or diagnostic tool. To increase the control of the labeling, conjugates based on maleimides, a common thiol-reactive reagent, are utilized. However, these have been shown to undergo premature cleavage due to exchange reactions with other free thiol groups, such as those present in serum albumin.^5^ In addition, cysteines are most commonly naturally paired, forming stabilizing disulfide bridges within the antibody, where the amount and location of the disulfides differ for different Immunoglobulin G (IgG) subclasses. Consequently, these different subclasses may be affected differently with regard to solubility and aggregation when these disulfides are broken.^8^ Furthermore, while one may achieve a higher control over the level of conjugation when utilizing cysteines compared to primary amine labeling, the exact level and site of conjugation can still not be decided in advance. There is also a recently developed method that utilizes glycans for attachment of the labeling group. This gives higher selectivity than targeting side chains of common amino acids, although not as high as the methods discussed below. To enable this, a partial deglycosylation with Endo S is performed where an artificial azide containing a galactose residue is bound to the remainder of the glycan group and can subsequently be utilized for labeling.^9^

To overcome the limitations of unspecific antibody conjugation, several methods have been developed with the aim of directing the conjugation to a specific location on the antibody, to avoid interference with the antibody target binding, as well as to gain control of the number of labels per antibody.

Although the main focus of this review will be on conjugation methods that are based on molecules with inherent affinity for the antibody scaffold, some of the directed conjugation methods that require modification of the actual antibody scaffold will be briefly discussed in the coming section.

## Site-Specific Conjugation Based on Antibody Modification

To avoid the heterogeneous labeling that is the result of traditionally used conjugation methods, a plethora of different methods have been developed with the aim of steering the conjugation to specific sites on the antibody. Many of these site-specific methods rely on engineering of the actual antibody itself prior to conjugation and is excellently reviewed elsewhere by, for example, Zhou et al.^10^ and Kline et al.^11^ among others. While this engineering approach might sound cost- and time-consuming, it may very well be worth it in otherwise costly processes such as ADC development, if it increases the therapeutic efficiency of the said antibody. Among these methods are, for example, those based on the engineering of cysteines as conjugation handles, which has been a successful strategy even though it might lead to stability issues due to the breaking of pre-existing disulfides.^6,12^ Other methods take advantage of nature’s way of carrying out site-specific conjugation by different enzymes, and these enzymatic reactions can be based either on recombinantly introduced recognition sites in the antibody sequence or on natural moieties such as glycans.

In addition to the 20 standard amino acids, there are also two additional amino acids, selenocysteine and pyrrolysine, that occur rarely, but naturally, in proteins that have been utilized for controlled conjugation of antibodies.^13−15^ Beyond the aforementioned natural, but rare, amino acids, there are also synthetically produced amino acids equipped with functional groups, known as noncanonical amino acids (ncAAs). These chemically orthogonal amino acids can be introduced recombinantly directly in the antibody sequence. Hundreds of these amino acids, with non-natural side chains, have been incorporated in proteins using both prokaryotic and eukaryotic production systems, and some of these ncAAs possess the unique chemical properties that make them suitable for conjugation.^16^ The most widely used strategy to introduce these amino acids *in vivo* is by amber suppression, a method that was first introduced by the lab of Peter Schultz almost two decades ago.^17^ Commonly used unnatural amino acids for conjugation are *p*-acetylphenylalanine (pAcF), *p*-azidophenylalanine (pAzF), *p*-azidomethylphenylalanine (pAMF), and an azide derivative of lysine (AzK).^18^

Many more methods exist that rely on antibody modification for conjugation, such as engineered tags,^19^ spycatcher,^20^ and split inteins.^21^ The focus of this review, however, is methods that do not rely on antibody modification, and these will be reviewed in more depth in the coming section.

## Affinity Ligands and Their Role in Site-Specific Conjugation of Unmodified Antibodies

**Figure 1:**
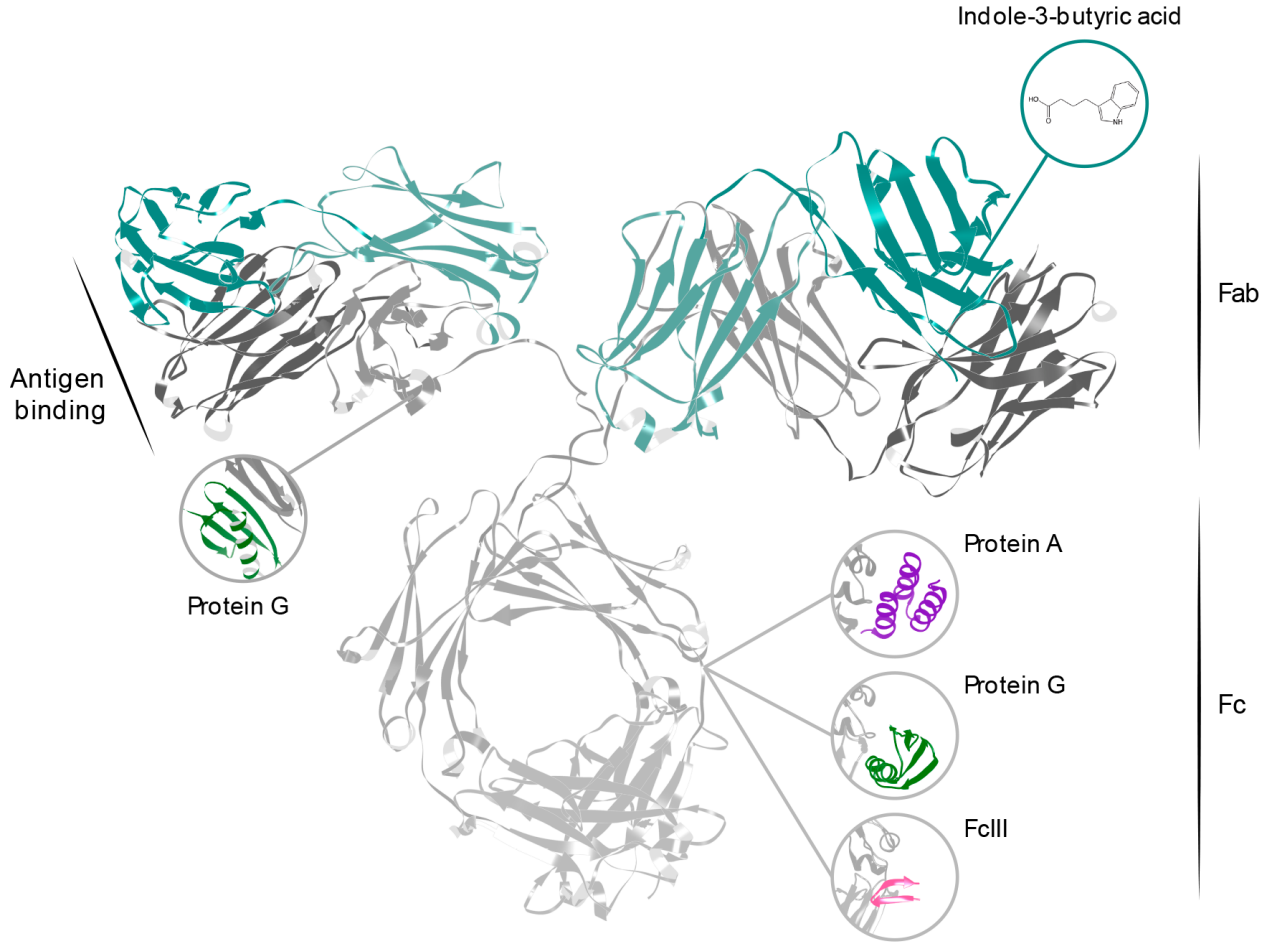
Structure of an IgG antibody (PDB ID:1IGT^22^) and a representation of the binding sites of the different affinity ligands reviewed in this paper. Protein A, Protein G, and FcIII all bind to the Fc fragment, between the two constant domains of the heavy chain (gray). Protein G interacts also with the heavy chain constant domain on the Fab fragment, while indole-3-butyric acid binds to the nucleotide binding site within the variable region of the Fab.

The methods described above are in many situations excellent for antibody conjugation and can be very useful when developing new ADCs. However, many antibodies already exist on the shelves and there is a need to be able to efficiently and site-specifically label these. Fortunately, there are many naturally existing, as well as synthetic, affinity ligands that already specifically recognize certain sites on antibodies, that are not part of the antibody’s paratope. An overview of these affinity ligands, and their binding sites, can be found in Figure 1.

Engineering of these affinity ligands to achieve covalent antibody modification present possibilities to site-specifically conjugate also off-the-shelf antibodies. Furthermore, these binders provide the means to label antibodies in complex surroundings such as cell culture supernatants or solutions containing stabilizing protein additives such as bovine serum albumin, that would interfere with antibody labeling when using traditional methods based on side chain conjugation. By utilizing a small molecule, peptide, or protein domain with affinity specifically for the antibody for conjugation, these types of antibody products can also be readily labeled in the solution in which they are provided. Table 1 shows an overview of the conjugation strategies described in this review.

**Table 1:** Overview of the Various Conjugation Strategies Described in This Review, Including Information on Conjugation Efficiencies and Antibody Subtype Specificity^a^

publication	affinity ligand	conjugation strategy	conjugation site	heavy chain conjugation efficiency^a^/antibody subtype specificity^b^
Jung et al.^23^	Protein G	Benzophenone/UV induced	Fc	50%/hIgG
Konrad et al.^24^	Protein A	Benzophenone/UV induced	Fc	ND/hIgG1, mIgG2, prIgG
Yu et al.^25^	Protein A	Benzophenone/UV induced	Fc	64%/mIgG1
Perols et al.^26^	Protein A	Benzophenone/UV induced	Fc	41%/hIgG1, 66%/mIgG1
Hui et al.^27^	Protein A	Benzophenone/UV induced	Fc	47%/hIgG1, 80%/mIgG3
Kanje et al.^28^	Protein G	Benzophenone/UV induced	Fc	90%/phIgG, 57%/prIgG
Kanje et al.^29^	Protein G	Benzophenone/UV induced	Fab	48%/mIgG1, 64%/mIgG2b, 43%/hIgG1, 58%/hIgG2, 52%/hIgG4
Hui et al.^30^	Protein G	Benzophenone/UV induced	Fc	90%/hIgG
Lee et al.^31^	Protein G	Photomethionine/UV induced	Fc	50%/hIgG, 50%/rIgG, 42%/gIgG
Ohata et al.^32^	Protein A	Catalyzation of alkyne-functionalized diazo modification	Fc	50%/phIgG, ND/hIgG, piIgG, rIgG, dIgG
Mori et al.^33^	Protein A	DSG/Chemical cross-linker	Fc	50%/hIgG1, 58%/mIgG2a
Yu et al.^34^	Protein A	Proximity induced	Fc	96%/hIgG1, 99%/hIgG2, 99%/mIgG1, 91%/mIgG2a, 99%/mIgG2b
Park et al.^35^	FcIII peptide	Benzophenone/UV induced	Fc	50%/hIG1^c^
Vance et al.^36^	FcIII peptide	Benzophenone/UV induced	Fc	95%/hIgG1
Kishimoto et al.^37^	FcIII peptide	DSG/Chemical cross-linker	Fc	100%/hIgG1
Alves et al.^38^	Indole-3-butyric acid	UV induced	Fab	62%/mIgG1

a Efficiencies are expressed as a mean value in the pool of conjugated antibodies where 100% corresponds to full occupancy of both binding sites, i.e. two labels per antibody.

b ND = Not disclosed, d = dog, g = goat, h = human, m = mouse, pi = pig, r = rabbit, p = polyclonal.

c Intentional, targeting monoconjugated antibodies.

### Conjugation Methods Based on Staphylococcal Protein A and Streptococcal Protein G

**Figure 2:**
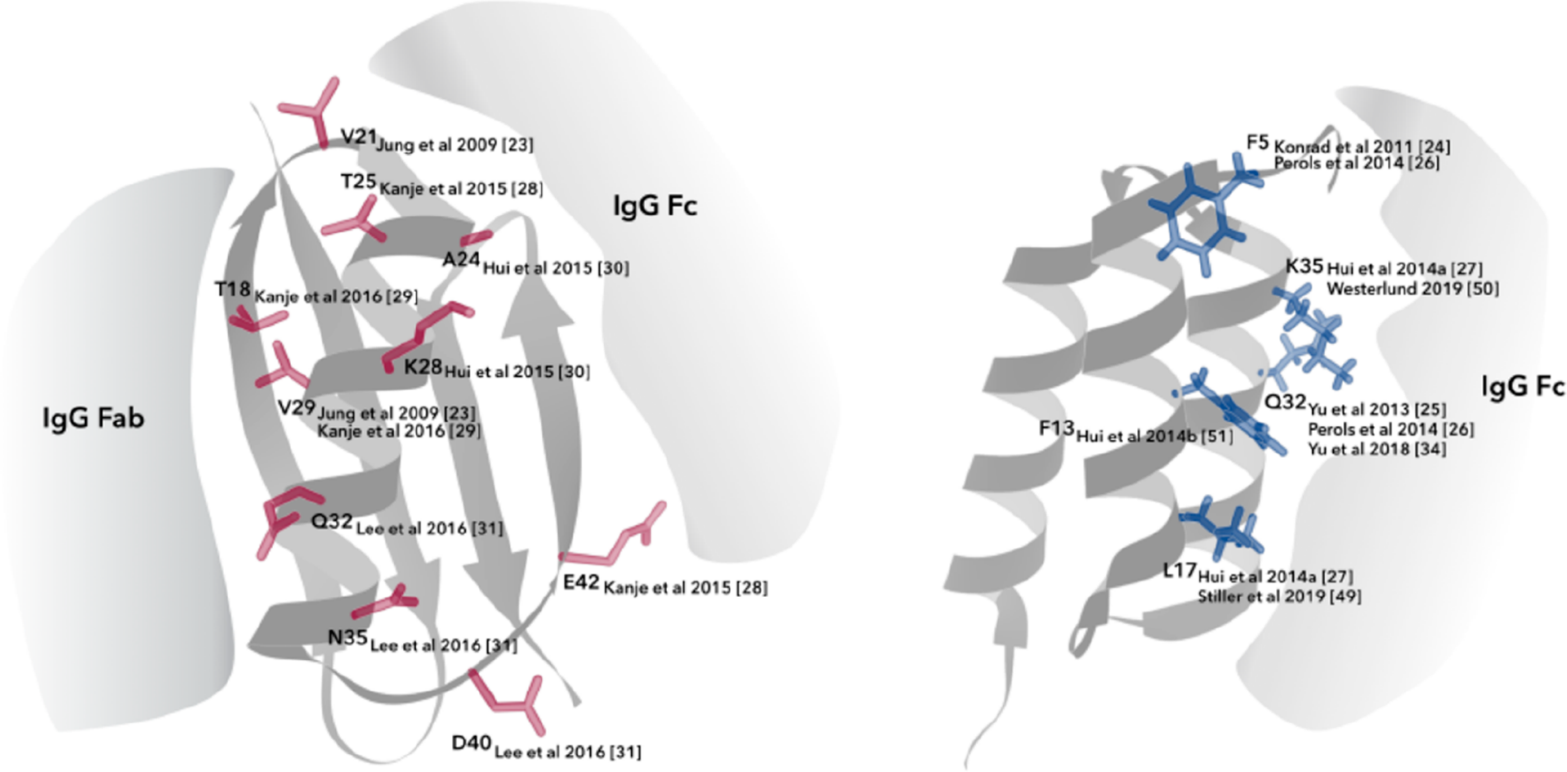
Amino acid positions utilized for antibody conjugation in domains of Protein G (left) and Protein A (right).

Protein A and Protein G are naturally occurring bacterial surface proteins with inherent affinity toward the conserved parts of IgG antibodies. Protein A comes from *Staphylococcus aureus* and consists of five highly homologous, small helical antibody binding domains with affinity mainly to the fragment crystallizable (Fc) part of IgG, but also to the V_H_ domain of human V_H_3 family antibodies.^39,40^ Protein G stems from group C and G Streptococci and is a multidomain protein that contains up to three highly homologous small β-sheet domains with an α-helix stacked on top, with affinity for both the Fc and the constant part of the fragment antigen binding (Fab) on the heavy chain C_H_1 domain.^41,42^ With their inherent affinity toward the conserved parts of IgG from many species and subclasses,^43^ these small bacterial domains are particularly suitable for directed antibody conjugation and labeling at specific sites that do not interfere with the antibody binding region. Figure 2 presents ribbon structure representations of protein domains from Protein A and G and different amino acid positions that have been utilized for antibody conjugation purposes, presented in further detail below.

### Benzophenone-Based Conjugation

**Figure 3:**
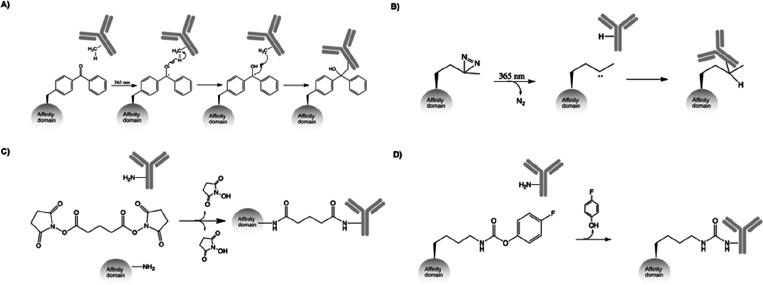
Reaction schemes of key conjugation methods reviewed in the text. (A) Benzophenone-based conjugation. (B) pMet based conjugation. (C) Disuccinimidyl glutarate (DSG) based conjugation. (D) 4-Fluorophenyl carbamate lysine based (pClick) conjugation.

In order to use these small protein domains for antibody labeling, one common strategy has been to introduce a benzophenone molecule to the Protein A or G domain. Benzophenones are UV-inducible at long-wavelength 365 nm light, which is generally considered to be harmless to proteins, and can covalently cross-link to nearby amino acids after activation or relax back to their initial state (Figure 3A).^44^ Normally, the benzophenone is introduced in or in proximity to the protein–antibody binding site, and covalently cross-links the antibody at the interaction interface. In the following examples, this results in cross-linking to the IgG heavy chain, at either the Fc or the Fab fragment.

First to publish this concept for antibody conjugation were Jung et al., who coupled the benzophenone to a Protein G C3 domain at positions 21 and 29, by mutating these to cysteine and conjugating the benzophenone, coupled to a flexible linker, to the protein domain using maleimide chemistry. Moreover, a N37Y mutation was introduced to abolish the domain’s natural affinity for Fab, to ensure that the conjugation took place only at the Fc fragment. The study showed that >50% of the antibodies had one or two Protein G domains covalently linked. Further, the cross-linking domain was equipped with an N-terminal biotinylation peptide that could be used for attachment to streptavidin-coated surfaces. Site-specifically conjugated antibodies were used for immobilization on glass surfaces and small particles, and it was shown that this directed immobilization provided a more efficient antibody–target binding compared to randomly biotinylated immobilized antibodies.^23^

Similar approaches have subsequently been used in multiple publications for both Protein A and Protein G domains to further increase cross-linking efficiencies and expand the applicability to other antibody subclasses and fragments, using various methods to introduce the benzophenone group. Konrad et al. used the ncAA *p*-benzoyl-phenylalanine (BPA) and incorporated it at position 5 of the Protein A-derived Z domain^45^ using solid-phase peptide synthesis (SPPS) and added a C-terminal biotin to the domain for labeling purposes. This molecule was shown to covalently label human IgG1, mouse IgG2a, and polyclonal rabbit IgG at the Fc fragment.^24^ Yu et al. introduced a F5I mutation to the Z domain to increase its affinity to mouse IgG1 and used a maleimide benzophenone (MBP) to introduce the cross-linking molecule at position 32 in the Z domain, after mutating it to cysteine. This domain showed a 64% coupling efficiency to mouse IgG1 heavy chains, where 50% corresponds to one cross-linked moiety per antibody. An antibody biotinylated using this domain showed better binding to its antigen in a surface plasmon resonance (SPR) interaction compared to its randomly biotinylated counterpart when immobilized on a streptavidin surface.^25^ In a later publication, the Z_F5IQ32MBP_ molecule was fused both N- and C-terminally to split enzyme halves of β-lactamase, that were then site-specifically conjugated to two different antibodies binding to separate epitopes on the same protein. This enabled analyte detection via split-enzyme complementation in a dual-antibody assay.^46^ Perols et al. used SPPS to introduce BPA or benzoylbenzoic acid (BBA, a benzophenone attached to a flexible linker) at positions 5 and 32 in Z, where Z32BPA could cross-link 41% of human IgG1 heavy chains and Z5BBA cross-linked 66% of mouse IgG1 heavy chains.^47^ Further improving the cross-linking abilities of the Z domain, Hui et al. reported up to 80% cross-linking of mouse IgG3 heavy chains by introducing BPA at position 17 in the protein domain and 47% conjugation efficiency for human IgG1 with BPA at position 35.^27^ For this particular labeling domain, BPA was introduced in the backbone of the Z domain using the technique described in section 2, where an orthogonal tRNA/aminoacyl tRNA synthetase pair incorporates the ncAA in response to the amber stop codon.^48^ The protein was equipped with a C-terminal sortase tag that could be used for conjugation of functional handles such as biotin or fluorophores to the labeling domain, and subsequently to antibodies.^27^ The Z domain with BPA at position 17 has later been used to conjugate DNA to antibodies that were used in an emulsion PCR setup to enable very sensitive protein detection.^49^ A Z domain with MBP coupled to a cysteine introduced at position 35 has been used for pretargeting in radionuclide imaging by conjugation of a peptide nucleic acid probe to said Z domain using a C-terminal sortase tag, and then conjugating the domain to the human epidermal growth factor receptor 2 (HER2)-binding antibody trastuzumab.^50^ Further, Hui et al. showed covalent attachment of antibodies to nanoparticles using a Z domain with BPA at position 13 where an azide containing peptide was fused to the Z domain via intein-mediated expressed protein ligation, which enabled cross-linked antibodies to be conjugated to nanoparticles via click chemistry.^51^

As for Protein G, apart from the initial study described by Jung et al., Kanje and Hober developed a domain for Fc conjugation containing BPA at positions 25 and 42 of the C2 domain, which showed cross-linking to 90% of polyclonal human IgG heavy chains and 57% of rabbit polyclonal IgG heavy chains. Human antibodies biotinylated with this domain showed a higher binding response when immobilized on a streptavidin surface in an SPR setup, compared to the same antibody that was randomly labeled using NHS-chemistry, and could also successfully be used for detection in an ELISA experiment.^28^ From the same research group, a C2 domain with abolished Fc binding, through the N35W and D40T mutations, was used for labeling of antibodies site-specifically at the Fab fragment. Introducing BPA at position 18 enabled 48–64% cross-linking to mouse IgG heavy chains depending on subclass, while BPA at position 29 provided 43–58% cross-linking to human IgG heavy chains depending on subclass. It was further shown that this Fab labeling agent could be combined with the Fc labeling domain to increase the signal in the ELISA setup used in the prior publication, and also that human antibodies labeled with these domains, either at Fc or at Fab, could still induce antibody-dependent cellular cytotoxicity.^29^ The Fab labeling domain was later also used in a multiplex imaging Western Blot setup using laser ablation inductively coupled plasma mass spectrometry (LA-ICP-MS) by site-specifically labeling mouse monoclonal antibodies with lanthanide metals.^52^ Another research group, led by professor Tsourkas, published a Protein G domain for Fc cross-linking with BPA at position 24 for mouse, rat, and rabbit IgG labeling or at position 28 for human IgG conjugation. That domain could also covalently label up to 90% of human IgG heavy chains. Similarly to their previously published Z labeling domain, discussed above, their Protein G labeling domain contains a C-terminal sortase tag allowing for labeling with different reactive groups.^30^ This labeling protein has been used for oligonucleotide conjugation to antibodies for incorporation to DNA nanostructures^53^ and for multiplexed cellular targeting.^54^ Further, site-specific conjugation of luciferase and fluorescent proteins to antibodies have been accomplished.^55^

Benzophenones have thus successfully been used in multiple variants of Protein A and G domains for site-specific conjugation to antibodies, at both the Fc and Fab fragment. The antibodies have been labeled with a plethora of different reporter molecules, and the conjugation efficiencies have constantly increased, leaving the scientific community with a vast variety of domains to modify their antibodies at specific sites with the desired number of labels, i.e. one, two, or three, depending on conjugation efficiency and labeling molecule combination. These methods provide a straightforward and mild way to site specifically label off the shelf antibodies in virtually any lab with access to a 365 nm UV light. With the improved labeling efficiencies, most of the published methods now make sure that on average at least one site on each antibody is labeled. Drawbacks include risk for immunogenicity in cases where the antibody is to be used *in vivo* as the protein domains, although small compared to the antibody, are still around 60 amino acids in size and foreign to the body. Furthermore, the labeled antibody pool would still be heterogeneous, normally consisting of a mix of antibodies with either zero, one, or two labels. Additionally, as the antibody domains used for labeling have a high natural affinity for the antibody, it can be a tedious and chemically harsh task to remove those domains that have not covalently reacted with the antibody.

### Other Conjugation Strategies

Other endeavors have utilized Protein A and G domains for antibody conjugation but exploited different strategies for cross-linking than the benzophenone approach. Lee et al. used the photoactivable ncAA photomethionine (pMet) to cross-link antibodies using 365 nm UV light (Figure 3B). The C3 domain of Protein G was used, and pMet was introduced at positions 32, 35 and 40 using amber suppression expression together with an N37R mutation to abolish Fab binding. The protein domain having pMet in all three positions could cross-link human and rabbit antibodies to 50% and was used to immobilize antibodies in a directed fashion on agarose beads and glass slides.^31^ pMet has the advantage of being smaller than the bulky benzophenone molecule, which could possibly lower the impact of exchanging an amino acid in or near the protein domain’s binding site.

Using a minimized Z domain of only 33 amino acids, containing only the 2 binding helices, developed by Braisted and Wells^56^ as its base protein, Mori et al. constructed a chemical conjugation affinity peptide (CCAP) with the introduced cross-linker disuccinimidyl glutarate (DSG) (Figure 3C) that could cross-link to a specific lysine residue in the Fc fragment. The domain was produced using SPPS and modified to exclude its original cysteines. It included a C-terminal azide-lysine residue that could be used for click-chemistry to attach functional molecules such as biotin to the CCAP. The domain was further modified with DSG in its N-terminus and used for cross-linking to human IgG1 and mouse IgG2a which resulted in 50% and 58% heavy chain modification, respectively. However, to obtain optimal labeling using this domain, the pH of the reaction needs to be lowered to 5.5, which may not be optimal for all antibodies. Mouse anti-IgE antibodies labeled by this domain showed twice as high sensitivity in a sandwich ELISA setup when compared to the same antibody randomly biotinylated. The site-specifically conjugated antibody could also be used for dose-dependent IgE detection on streptavidin-coated latex beads in a reversed passive latex agglutination assay, which the same randomly biotinylated detection antibody could not.^33^

Also using conventional cross-linkers for antibody conjugation, Shroeder et al. preactivated Protein A and Protein G coated microtiter plates with the cross-linkers succinimidyl (4-iodoacetyl) aminobenzoate (SIAB) and sulfosuccinimidyl (4-iodoacetyl) aminobenzoate (sulfo-SIAB) bifunctional reagents before adding antibody that could site-specifically cross-link at the Fc to the immobilized proteins on the plate, after which non-cross-linked antibodies could be washed away under acidic conditions. The specific and directed immobilization gave higher signals compared to undirected immobilization. Moreover, it enabled the possibility to immobilize antibodies directly from a crude supernatant.^57^

Yu et al. presented another proximity induced site-specific conjugation of the B domain, of Protein A, to Fc, requiring no UV or chemical treatment, which was denoted pClick. The ncAA 4-fluorophenyl carbamate lysine that can react with nearby lysines (Figure 3D) was introduced at position 25 of the B domain and could cross-link 91–99% of human IgG1 and 2 and mouse IgG1, 2a, and 2b heavy chains. While this method provides very efficient labeling, the pClick reaction is rather slow, as it requires 2 days of incubation for optimal results. This domain was used to label a HER2-targeting antibody with fluorescein which was successfully used for detection of HER2 on cell surfaces.^34^

### Conjugation Methods Based on the FcIII Peptide

Although many affinity reagents used for conjugation are protein domains like those mentioned above, or even larger macromolecules, there are examples demonstrating that only a short stretch of amino acids can be enough to ensure a large enough contact area for highly selective binding. As a smaller alternative to the naturally occurring Fc-binding proteins A and G, a short cyclic peptide binding to the same region of IgG has also been reported. DeLano et al. isolated two peptides, FcI and FcII, from a phage library of 4 × 10^9^ different disulfide-induced cyclic peptides of the form X*i*CX*j*CX*k* (where C is a cysteine, X is a random amino acid, and *i*+*j*+*k* = 18). These peptides were then further matured by construction of new libraries that resulted in the 13-mer peptide FcIII (DCAWHLGELVWCT), binding to the Fc region with a strength of approximately half of Protein A and G, which both have equilibrium binding constants (*K*_D_) around 10 nM. The same publication also includes an X-ray structure of the complex between the FcIII peptide and the IgG Fc fragment (PDB ID: 1DN2). The protein structure shows that, despite the peptide having a completely different structure than other natural Fc-binding proteins such as Protein A, Protein G, the neonatal Fc receptor, and the rheumatoid factor, it does indeed overlap the same Fc binding site, namely, the interface between the C_H_2 and C_H_3 domains. The formation of a β-bulge conformation allows 8 of the amino acids to form interactions with the Fc, covering a binding area of 650 Å, almost the same size as for the much larger natural binding proteins. Many of the interactions of the individual residues also share common features between the peptide and the naturally occurring Fc-binding proteins.^58^ An 80-fold improvement of the affinity was later achieved by Dias et al. by transplanting the FcIII peptide onto a stabilizing d-Pro-l-Pro template creating a backbone cyclic peptidomimetic which stabilized the fold, previously only held together by the disulfide.^59^

The FcIII peptide has found several different areas of applications, including that of fusion to a fluorescent model protein to demonstrate improvement in circulatory half-life,^60^ as well as immobilization on a sepharose resin for high-affinity purification of antibodies.^61^ However, it took almost two decades since the first discovery of the peptide, before it was used for antibody conjugation. Park et al.^35^ and, shortly thereafter, Vance et al.^36^ both demonstrated conjugation of a payload to antibodies through the introduction of the photoactivable ncAA BPA into different positions of the peptide. Both groups drew the conclusion that optimal conjugation results were achieved when introducing BPA in position 10. While Park et al. focused on monoconjugated antibodies, Vance et al. achieved a drug-to-antibody-ratio of 1.9 which corresponds to approximately 95% conjugation efficiency.

A closely related peptide, the IgG-BP, differing from FcIII only by the W6Y and L8R substitutions, have also been utilized for site-specific attachment. By introducing a lysine residue in position 8 of this peptide and thereafter cross-linking it to the nearby Lys248 of an IgG-Fc through the cross-linker DSG, this peptide has been used to prepare both an ADC by conjugation of the maytansine derivative DM1 and a bispecific antibody by conjugation to a nanobody.^62^ This same peptide, together with other previously known peptides such as the original FcIII and the minimized Z domain described above, also forms the basis of the AJICAP technology, described by Yamada et al. This technology is based on the functionalization of native antibodies with thiol groups through the use of these affinity reagents, which then allows for further conjugation of cytotoxic payloads.^63^ The same group later also describes the use of the AJICAP technology for gram-scale synthesis of stable and homogeneous ADCs.^64^

Taken together, these examples show how nonmodified antibodies can be efficiently site-specifically conjugated by utilizing a peptide as small as 13 amino acids. It should be noted that the function of the FcIII peptide relies on formation of a disulfide and that care must be taken to ensure the desired redox state. This fact also precludes the introduction of additional thiol groups to be used as functional handles. However, since the peptide is so small, it can be easily produced by a chemical synthesis approach, which in turn entails a simple way of introducing many other non-natural functional groups.

### Conjugation Methods Based on the Nucleotide Binding Site

A new, unconventional binding site in the variable part of antibodies was described by Rajagopalan et al. in 1996. The new binding site, later denoted NBS (Nucleotide Binding Site), was shown to bind adenosine triphosphate (ATP) with an apparent *K*_D_ of 75 μM.^65^ In 1997, the same research group was able to show that they could utilize that binding site for directed photolabeling with a biotin. The functional group used for biotinylation was an azidoadenosine decorated with a biotin, where the azido group, photolyzed with 254 nm, a wavelength which could potentially affect the antibody more than the 365 nm used for benzophenone photolabeling, was responsible for the covalent attachment.^66^ Later, in 2013, this concept was further developed by the use of a new ligand with higher affinity for the NBS, namely, indole-3-butyric acid (IBA).^38^ The labeling procedure regarding buffer composition, ligand concentration, and UV energy was optimized to reach a high degree of specific labeling. By utilizing modified IBA, the authors were able to conjugate antibodies with a number of different groups including biotin, fluorescein isothiocyanate (FITC), a peptide, and the chemotherapeutic paclitaxel. Furthermore, the same labeling method has also been utilized for directed solid-phase attachment of antibodies to streptavidin-coated surfaces.^38^ In order to develop a method for antigen detection, the group of Ueda utilized the NBS to conjugate an antibody with a fluorophore. Here, the proximity with the antigen binding site could be utilized, since the fluorescence of the conjugated molecule was shown to be quenched by the free paratope, meaning that the fluorophore was shown to fluoresce only when the antigen was bound.^67^ This proximity has also been utilized to inhibit IgE clustering on mast cells by blocking the paratope through covalent attachment of an optimized molecule to the NBS that simultaneously binds to the paratope of the IgE.^68^ In conclusion, NBS provides an interesting site for antibody conjugation on the Fab fragment, which can be utilized for conjugation using small molecules rather than entire peptides or proteins. Drawbacks for the method include the use of 254 nm UV light, which can be detrimental to the antibody, and the need for a very pure antibody sample, which requires removal of preservatives and stabilizers in the sample prior to labeling.

### Traceless Conjugation Strategies

The conjugation methods described above all rely on the covalent attachment of an affinity ligand along with the probe of interest onto the antibody. However, other methods exist where the affinity ligand functions merely to bring a catalyst in close proximity to the conjugation site for a completely traceless conjugation of the probe alone onto the antibody. This might be particularly interesting for many therapeutic applications where, for example, immunogenicity of the affinity ligand could be a potential concern.

Recently, Yu et al.^69^ presented an elegant approach for traceless labeling of human IgG1, where a mutated Sortase A combined with an antibody binding domain, a Protein G domain, or a calcium-dependent Z domain,^70^ could facilitate labeling of five specific lysine residues on the antibody heavy chain and one on the light chain with a labeling agent attached to a LPETG motif. The average number of labels per antibody was determined to 2.3 or 2.9 depending on the analysis method used, and the labeling did not interfere with the antibody’s ability to bind its antigen, the neonatal Fc receptor (FcRn), or the Fc gamma receptor I (FcγRI). After labeling, the Sortase A-antibody binding domain could be removed from the antibody with an acidic wash or calcium removal by EDTA. An obvious upside to the traceless strategies is that it does not leave a bulky protein domain attached as the bridge between the antibody as its label. However, while the method described here was shown to attach the label to five specific lysines on the antibody, it appears to be less homogeneous in regard to the labeling site compared to the methods based on affinity domains with one well-known binding site on the antibody.

Using the previously mentioned minimized Z domain,^56^ Ohata et al. further developed this domain by introducing three di-rhodium centers at residues E3, E11, and E20 of the domain. The rhodium-containing domain was then used for catalysis of alkyne-functionalized diazo modification of IgG, which was not possible in the presence of rhodium complexes on their own. The molecule was produced using SPPS and could catalyze modification of antibodies by attachment of the chemical handle to position N79 in the Fc fragment. An alkyne functionalized human IgG1 antibody was then shown to conjugate fluorophores, and could also conjugate drugs in order to make an ADC.^32^

Another example utilizes the already described FcIII peptide to form stable antibody–DNA conjugates. Here, the FcIII peptide functions to guide a template DNA to the binding site, while a complementary DNA strand is conjugated to the Fc through a benzaldehyde moiety reacting with a nearby lysine side chain, without the involvement of the actual peptide. Authors of this study used these DNA-conjugated antibodies to assemble IgG pentamers around a core DNA motif, mimicking the natural IgM structure.^71^

A somewhat analogous strategy with the same aim of conjugating DNA to the antibody utilizes a class of affinity ligands known as Aptamers, which are single-stranded oligonucleotides that can bind specifically to a target molecule. To achieve a traceless conjugation, the Aptamer is decorated with an oligonucleotide linker that, following binding of the target molecule, can hybridize to a partially complementary reacting oligonucleotide containing an activated carboxyl residue that reacts with a nearby amine residue at the specific site. The template Aptamer can then be removed by addition of a fully complementary cDNA. Cui et al. have demonstrated this method for several biologically relevant proteins and used a particular Aptamer specific for the human IgG Fc domain to create a traceless antibody–DNA conjugate with high specificity.^72^

### Noncovalent Conjugation Strategies

The methods described above, traceless or not, are all based on covalent conjugation of an antibody payload to ensure stable conjugates in complex environments. In contrast to these methods, a few examples exist that instead rely solely on a noncovalent, high-affinity, selective interaction.

The first example of noncovalent antibody conjugation is based on the same Z domain as the covalent methods mentioned above. To achieve a high enough affinity for the stable interaction, Zhou et al. coupled two Z domains via a long enough flexible linker so that each domain of the protein could bind the two binding sites for Z on one Fc fragment simultaneously. This improved the affinity for Fc compared to the single Z domain and rendered a molecule with up to 84-fold slower off-rate compared to the Z domain on its own. The Z-linker-Z domain was then circularized using a split intein approach and termed lasso. It was also equipped with an N-terminal biotinylation tag as well as a cysteine for additional reporter conjugation. The lasso was used as a capturing agent in an ELISA and shown to lower the limit of detection compared to an immobilized secondary Fab fragment by 12-fold and also, when conjugated to a fluorophore, used as a detecting agent in a confocal microscopy cell detection assay.^73^

Another method that does not rely on covalent conjugation is the method known as multivalent and affinity-guided antibody empowerment technology (MAGNET). Here, Gupta et al. report on a computational approach used to simulate and design affinity ligands that bind strongly to certain conserved residues of an IgG1 antibody. As a template for the simulations, the well-known affinity molecule 4-mercaptoethylpyridine (MEP) coupled to two different payloads was used. The resulting MAGNET linkers were shown to rapidly associate to IgG by simply mixing the two molecules together. The authors also demonstrate that, despite the interaction being noncovalent, the conjugated antibodies were stable in both mouse and human plasma for 14 days. In addition, the efficacy of the conjugate was demonstrated using an *in vivo* xenograft model of human lung adenocarcinoma.^74^

While very promising and obviously stable, some applications may still require covalent attachment of the label to ensure little to no label loss, in particular, when using detection antibodies in harsher reaction conditions for which these methods may not be optimal.

## Conclusions and Future Outlook

Presented in this review are methods for conjugation of reporter molecules and therapeutics to antibodies in a site-specific and controlled manner. These methods are based on molecules with inherent affinity for parts of the antibody that are outside of the paratope and provide the means to label any off-the-shelf antibody without modifying it first. These methods are important new tools, for making both detection antibodies and ADCs. Many of the aforementioned examples have shown the benefits of site-specific labeling compared to the traditional random conjugation methods that rely on side chain modifications, which may impair both the antibody structure and binding site. The possibility of using these methods to label antibodies even when present in complex mixtures makes them broadly applicable to many different types of samples, possibly even including cells and tissues *in vivo*. Thus, these antibody conjugation molecules are very important tools available for the many research fields utilizing antibodies. We believe that the use of these affinity ligands will continue to contribute to the field of antibody conjugation and ADC development in the future as they will continue to develop. Future endeavors should focus on improving the labeling efficiency as well as stability and to evaluate different combinations of ligands to increase the number of labels per antibody. This will be important both for development of ADCs with a higher drug-to-antibody-ratio (DAR) and for signal amplification during detection, to achieve more robust tools and more potent drugs. The traceless approaches mentioned above, where the affinity ligand works only as a catalyst holds great potential for simple and nonimmunogenic conjugates.
